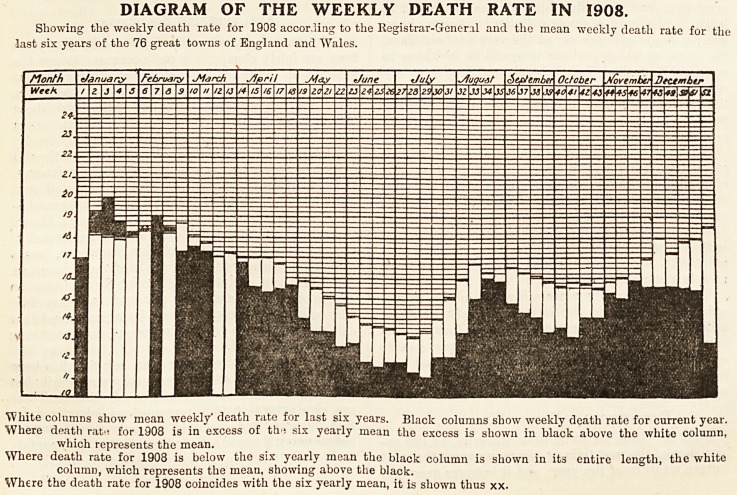# A Healthy Year

**Published:** 1909-01-16

**Authors:** 


### PUBLIC HEALTH AND HYGIENE.

### A HEALTHY YEAR.

THE diagram, published below, presents a remarkable feature of the pathodermic constitution of the year just closed. The marked deviation from the mean death-rate for the last six years of the seventy-six great towns of the Registrar-General, in the direction of diminished mortality, is indeed a most striking fact.

In our issue of January 18 last year we were able to exhibit a departure in many respects similar from the corresponding mean death-rate of the previous quinquennium. The year 1908, however, is marked by a more constant conformity to the mean curve than characterised the year 1907. The death-rate is almost uniformly lower throughout the year; 1908 in fact is judged, by its mortality rate; one of the healthiest years on record. So considerable a reduction of mortality can hardly be ascribed to any of the slowly progressive changes which make for the gradual improvement of the public health. There can be no question that in large measure it is to be ascribed to the anomalous meteorological conditions that have characterised the seasons during the last two years. Zymotic diarrhœa, which is so fatal to infants in the late summer and early autumn months, has been deprived of its harvest, so to speak, during the last two years by the unseasonable weather which has marked the period of its seasonal prevalence. The summer of 1908, like that of 1907, was abnormally cold and wet, and there is no doubt that a large number of infants have survived an extremely fatal period of infantile life directly as the result of this accident of climate. The later months of 1908, on the other hand, were unseasonably warm and mild, and this again has been responsible for a great reduction in the number of deaths, which are the invariable concomitant of cold and inclement weather in these islands. The association of a green yule with a fat kirkyard is one of ideas and not of fact. No doubt the sense of well-being is keener in the cold, dry, frosty weather of a severe winter than in the comparatively warm, damp, muggy climate of a mild one, but the enjoyment by the vigorous of a sense of health must not be interpreted as being synonymous with a healthy climatic environment. For those who are strong and robust, severe weather may be stimulating and invigorating, but to the delicate, the aged, the underfed, underclothed, and badly housed poor, low temperatures generally mean high death-rates.

An investigation of all the causes which have marked the year as one of exceptionally low mortality would involve a complex analysis, the data for which are not available; but the very remarkable picture presented by the diagram may safely be counted as some compensation for the vexatious vagaries of climate and season in the year through which we have just passed.

### DIAGRAM OF THE WEEKLY DEATH RATE IN 1908.

Showing the weekly death rate for 1908 according to the Registrar-General and the mean weekly death rate for the last six years of the 76 great towns of England and Wales.

White columns show mean weekly death rate for last six years. Black columns show weekly death rate for current year.
Where death rate for 1908 is in excess of the six yearly mean the excess is shown in black above the white column, which represents the mean.
Where death rate for 1908 is below the six yearly mean the black column is shown in its entire length, the white column, which represents the mean, showing above the black.
Where the death rate for 1908 coincides with the six yearly mean, it is shown thus xx.

## Figures and Tables

**Figure f1:**